# Supplementary material for: Health knowledge, health behaviors and attitudes during pandemic emergencies: A systematic review
Source: PLoS One. 2021 Sep 7;16(9):e0256731. doi: 10.1371/journal.pone.0256731 (PMC8423234; doi:10.1371/journal.pone.0256731)
Supplement: S1 Table — (DOCX) [file pone.0256731.s001.docx]

**S1 Table. Inclusion and exclusion criteria for article selection**

| Criteria | Inclusion | Exclusion |
| --- | --- | --- |
| Health Knowledge Measurement | Health knowledge is evaluated through information elements such as: definition, transmission, etiology, prevention, risk factors, symptoms or treatment.  Health knowledge is measured as a self-report measure at the individual, group or population level. | Health knowledge is not associated with a pandemic context.  Health knowledge is evaluated as general knowledge about pandemics. |
| Behavior measurement | Behaviors are measured through hygiene practices, self-protection measures, medical aid or social restriction.  Behavior is measured as a self-report at the individual, group, or population level. | Behaviors are not behavioral responses associated with pandemics.  Behaviors are post-pandemic practices. |
| Attitude measurement | Attitudes are evaluated through valuations or perceptions based on objective information associated with prevention or health measures.  Attitudes are evaluated as a self-report at the individual, group or population level. | Attitudes are not associated with pandemics.  Attitudes are generated post pandemics. |
| Design | The study is empirical and quantitative in approach.  The study was carried out under an experimental design (Randomized Controlled Trials): observational (cohort or case-control), cross-sectional or longitudinal studies. | The study exclusively reports qualitative data.  The study is a systematic review or meta-analysis. |
| Sample | Participants are at least 18 years old.  The sample represents population cohorts from community, clinical or academic contexts. | Exclusively includes children or adolescents’ samples. |
| Outcome | The result is an association measure between health knowledge, behaviors and attitudes.  The results may be some of these:  1. Health knowledge has a effect on the behavior and attitudes adoption.  2. Health knowledge is a predictor, mediator or moderator factor.  3. Health knowledge is correlated with behavior or attitudes. | Not enough original data provided.  Merely descriptive analysis without reporting effects or association measures. |
| Time | Studies published between January 2009 and June 2020. |  |
