# Supplementary material for: Health knowledge, health behaviors and attitudes during pandemic emergencies: A systematic review
Source: PLoS One. 2021 Sep 7;16(9):e0256731. doi: 10.1371/journal.pone.0256731 (PMC8423234; doi:10.1371/journal.pone.0256731)
Supplement: S2 Table — (DOCX) [file pone.0256731.s002.docx]

**S2 Table. Quality assessment of included studies**

|  |  | Selection | | | | Comparability | Outcome | |  |  |
| --- | --- | --- | --- | --- | --- | --- | --- | --- | --- | --- |
| Study (First author and year) | Study Design | Representativeness of the sample | Sample size | Non-respondents | Ascertainment of exposure | Based on design and analysis | Assessment of outcome | Statistical Test | Total | Risk of bias |
| Almutairi et al., 2015 | Cross-sectional | / | / | / | * | ** | * | * | 5 | High |
| Askarian et al., 2013 | Cross-sectional | / | * | / | ** | ** | * | * | 7 | Average |
| Etingen et al., 2013 | Cross-sectional | / | * | / | * | ** | * | * | 6 | High |
| Ho et alk., 2013 | Cross-sectional | * | * | / | ** | ** | * | * | 8 | Average |
| Keller et al., 2014 | Cross-sectional | / | / | / | ** | ** | * | * | 6 | High |
| Krishnappa et al., 2020 | Cross-sectional | / | / | * | ** | ** | * | * | 7 | Average |
| Liao et al., 2010 | Cross-sectional | * | * | / | ** | ** | * | * | 8 | Average |
| Lin et al., 2011 | Cross-sectional | / | * | * | * | ** | * | * | 7 | Average |
| Nabil et al., 2010 | Cross-sectional | * | * | / | * | ** | * | * | 7 | Average |
| Ping et al., 2011 | Cross-sectional | / | / | * | ** | ** | * | * | 7 | Average |
| Rahman et al., 2020, | Cross-sectional | / | / | / | ** | ** | * | * | 6 | High |
| Yap et al., 2010 | Cross-sectional | * | * | / | * | ** | * | * | 7 | Average |
| Zhang et al., 2020 | Cross-sectional | / | * | / | * | ** | * | * | 6 | High |
