# Supplementary material for: Health knowledge, health behaviors and attitudes during pandemic emergencies: A systematic review
Source: PLoS One. 2021 Sep 7;16(9):e0256731. doi: 10.1371/journal.pone.0256731 (PMC8423234; doi:10.1371/journal.pone.0256731)
Supplement: S3 Table — (DOCX) [file pone.0256731.s003.docx]

**S3 Table. Operationalization of the health knowledge measurement**

| Study (First author and year) | Health Knowledge Measures | | | | | | | | |
| --- | --- | --- | --- | --- | --- | --- | --- | --- | --- |
|  | Etiology | Definition | Transmission | Symptomatology | Infection severity | Prevention | Comunicability period | Vaccine availability | Incubation period |
| Almutairi et al., 2015 | x | x | x | x |  | x | x | x | x |
| Askarian et al., 2013 | x |  | x | x | x | x |  | x | x |
| Etingen et al., 2013 |  |  | x |  |  | x |  |  |  |
| Ho et alk., 2013 |  |  | x | x | x | x |  | x |  |
| Liao et al., 2010 | x |  | x |  |  | x |  |  |  |
| Lin et al., 2011 |  |  | x |  |  |  |  |  |  |
| Keller et al., 2014 |  |  | x | x |  | x |  |  |  |
| Krishnappa et al., 2020 |  | x | x | x |  | x |  | x |  |
| Nabil et al., 2010 |  |  | x | x | x | x |  |  |  |
| Ping et al., 2011 |  |  | x | x | x |  |  |  |  |
| Rahman et al., 2020, |  |  | x | x |  | x |  |  |  |
| Yap et al., 2010 |  | x | x | x |  | x |  |  |  |
| Zhang et al., 2020 |  | x | x |  |  | x |  |  |  |
| *TOTAL* | *4* | *4* | *12* | *9* | *4* | *11* | *1* | *4* | *2* |
