# Supplementary material for: Health knowledge, health behaviors and attitudes during pandemic emergencies: A systematic review
Source: PLoS One. 2021 Sep 7;16(9):e0256731. doi: 10.1371/journal.pone.0256731 (PMC8423234; doi:10.1371/journal.pone.0256731)
Supplement: S2 Appendix — (DOCX) [file pone.0256731.s005.docx]

**S2 Appendix. Search criteria**

The table presents the search strategy for all bases. The controlled vocabulary browser was used for the search strategy MeSH (Medical Subject Headings).

| Table 1. Full Search criteria for electronic databases | | |
| --- | --- | --- |
| Keywords | **Numeration** | **Search criteria** |
| Health Awareness | 1. | MeSH descriptor Health Awareness (this term only) (Title/Abstract) |
|  | 2. | Awareness, Health |
|  | 3. | #1 OR #2 |
| Health Knowledge | 4. | MeSH descriptor Health Knowledge (this term only) (Title/Abstract) |
|  | 5. | knowledge, Health |
|  | 6. | #4 OR #5 |
|  | 7. | #3 OR #6 |
| Pandemic | 8. | MeSH descriptor Pandemic (this term only) (Title/Abstract) |
|  | 9. | MeSH descriptor H1N1 (this term only) (Title/Abstract) |
|  | 10. | MeSH descriptor SARS Virus (this term only) (Title/Abstract) |
|  | 11. | MeSH descriptor Influenza A virus (this term only) (Title/Abstract) |
|  | 12. | MeSH descriptor COVID-19 (this term only) (Title/Abstract) |
|  | 13. | #8 OR #9 OR #10 OR #11 OR #12 |
|  | 14. | #3 AND #6 AND #13 |
| Health Behavior | 15. | Health Behavior (this term only) (Title/Abstract) |
|  | 16. | Behavior, Health |
|  | 17 | #15 AND #16 |
| Attitude to Health | 18. | MeSH descriptor Health Attitudes (this term only) (Title/Abstract) |
|  | 19. | Attitude, Health |
|  | 20. | #18 OR #19 |
|  | 21. | #3 AND #6 AND #13 AND #17 AND #20 |

**APA**

1. **Any Field:** Health Awareness AND **Any Field:** Pandemic* OR **Any Field:** H1N1 OR **Any Field:** SARS OR **Any Field:** Influenza A virus OR **Any Field:** Covid-19 AND **Publication Type**: Journal AND **Age Group**: Adulthood (18 yrs. & older) AND **Year:** 2009 To 2020.
2. **Any Field:** Health Behavior AND **Any Field:** Pandemic* OR **Any Field:** H1N1 OR **Any Field:** SARS OR **Any Field:** Influenza A virus OR **Any Field:** Covid-19 AND **Publication Type**: Journal AND **Age Group**: Adulthood (18 yrs. & older) AND **Year:** 2009 To 2020.
3. **Any Field:** Attitude to Health AND **Any Field:** Pandemic* OR **Any Field:** H1N1 OR **Any Field:** SARS OR **Any Field:** Influenza A virus OR **Any Field:** Covid-19 AND **Publication Type**: Journal AND **Age Group**: Adulthood (18 yrs. & older) AND **Year:** 2009 To 2020.
4. **Any Field:** Health Knowledge AND **Any Field:** Pandemic* OR **Any Field:** H1N1 OR **Any Field:** SARS OR **Any Field:** Influenza A virus OR **Any Field:** Covid-19 AND **Publication Type**: Journal AND **Age Group**: Adulthood (18 yrs. & older) AND **Year:** 2009 To 2020.

**PubMed**

1. ("Health awareness" [MeSH Terms]) AND Pandemic [Title/Abstract]) OR SARS[Title]) OR H1N1[Title/Abstract]) OR COVID-19[Title/Abstract]) OR Influenza A virus [Title/Abstract]) Filters: published in the last 10 years
2. ("Attitude to health" [MeSH Terms]) AND Pandemic [Title/Abstract]) OR SARS[Title]) OR H1N1[Title/Abstract]) OR COVID-19[Title/Abstract]) OR Influenza A virus [Title/Abstract]) Filters: published in the last 10 years
3. ("Health Knowledge" [MeSH Terms]) AND Pandemic [Title/Abstract]) OR SARS[Title]) OR H1N1[Title/Abstract]) OR COVID-19[Title/Abstract]) OR Influenza A virus [Title/Abstract]) Filters: published in the last 10 years
4. ("Health Behavior" [MeSH Terms]) AND Pandemic [Title/Abstract]) OR SARS[Title]) OR H1N1[Title/Abstract]) OR COVID-19[Title/Abstract]) OR Influenza A virus [Title/Abstract]) Filters: published in the last 10 years
5. #1 AND #2 AND #3 AND #4

**Embase**

1. (“Health awareness” AND Pandemic* OR “influenza a virus (h1n1) OR SARS OR Covid-19) [2009-2020]/py AND [embase]/lim NOT ([embase)]/lim AND [medline]/lim) AND ([adult]/lim OR [aged]/lim OR [middle aged]/lim OR [young adult]/lim) AND (“article”/it OR “article in press”/it)
2. (“Attitude to health” AND Pandemic* OR “influenza a virus (h1n1) OR SARS OR Covid-19) [2009-2020]/py AND [embase]/lim NOT ([embase)]/lim AND [medline]/lim) AND ([adult]/lim OR [aged]/lim OR [middle aged]/lim OR [young adult]/lim) AND (“article”/it OR “article in press”/it)
3. (“Health Knowledge” AND Pandemic* OR “influenza a virus (h1n1) OR SARS OR Covid-19) [2009-2020]/py AND [embase]/lim NOT ([embase)]/lim AND [medline]/lim) AND ([adult]/lim OR [aged]/lim OR [middle aged]/lim OR [young adult]/lim) AND (“article”/it OR “article in press”/it)
4. (“Health Behavior” AND Pandemic* OR “influenza a virus (h1n1) OR SARS OR Covid-19) [2009-2020]/py AND [embase]/lim NOT ([embase)]/lim AND [medline]/lim) AND ([adult]/lim OR [aged]/lim OR [middle aged]/lim OR [young adult]/lim) AND (“article”/it OR “article in press”/it)

**Cochrane library**

1. “Health awareness” in ALL Text AND “Pandemic” in title Abstract Keyword OR Covid-19 in Tittle Abstract Keyword OR H1N1 in Tittle Abstract Keyword OR SARS in Tittle Abstract Keyword OR Influenza A virus in Tittle Abstract Keyword – in Trials (Word variations have been Searched)
2. “Health Behavior” in ALL Text AND “Pandemic” in title Abstract Keyword OR Covid-19 in Tittle Abstract Keyword OR H1N1 in Tittle Abstract Keyword OR SARS in Tittle Abstract Keyword OR Influenza A virus in Tittle Abstract Keyword – in Trials (Word variations have been Searched)
3. “Attitude to Health” in ALL Text AND “Pandemic” in title Abstract Keyword OR Covid-19 in Tittle Abstract Keyword OR H1N1 in Tittle Abstract Keyword OR SARS in Tittle Abstract Keyword OR Influenza A virus in Tittle Abstract Keyword – in Trials (Word variations have been Searched)
4. “Health Knowledge” in ALL Text AND “Pandemic” in title Abstract Keyword OR Covid-19 in Tittle Abstract Keyword OR H1N1 in Tittle Abstract Keyword OR SARS in Tittle Abstract Keyword OR Influenza A virus in Tittle Abstract Keyword – in Trials (Word variations have been Searched)
