# Supplementary material for: Health knowledge, health behaviors and attitudes during pandemic emergencies: A systematic review
Source: PLoS One. 2021 Sep 7;16(9):e0256731. doi: 10.1371/journal.pone.0256731 (PMC8423234; doi:10.1371/journal.pone.0256731)
Supplement: Attachment — Submitted filename: PONE-D-20-31471_reviewer.pdf [file pone.0256731.s006.pdf]

PLOS ONE

Effects of health knowledge in the health behaviors and attitudes adoption during pandemic emergencies: a rapid review

--Manuscript Draft--

**Manuscript Number:**	PONE-D-20-31471
**Article Type:**	Research Article
**Full Title:**	Effects of health knowledge in the health behaviors and attitudes adoption during pandemic emergencies: a rapid review
**Short Title:**	Health knowledge, health behaviors and attitudes during pandemics
**Corresponding Author:**	Fabio Alexis Rincon Uribe<br>Universidade Federal do Pará<br>Belém, PA BRAZIL
**Keywords:**	Health Awareness; Health Knowledge; Pandemic; Health Behavior; Health Attitudes
**Abstract:**	Background<br>People with good health knowledge present a conceptual and objective appropriation on general and specific health topics, increasing their probability to express health protection and prevention measures. The main objective of this study was to carry out a literature quick review about the effects of health knowledge on the adoption of health behaviors and attitudes in populations under pandemic emergencies.<br>Methods<br>A systematic review was performed according to the parameters of the PRISMA-P Group and the Cochrane method for rapid systematic reviews. Studies searches were performed in APA PsycNet, Embase, Cochrane Library and PubMed Central. Studies published between January 2009 and June 2020 and whose primary results reported a measure of interaction between health knowledge, health attitudes and behaviors in population groups during pandemics were included. A review protocol was recorded in PROSPERO (CRD42020183347).<br>Results<br>Out of a total of 5791 studies identified in the databases, 13 met the inclusion criteria. The included studies contain a population of 26099 adults, grouped into cohorts of health workers, university students, clinical patients, and the general population. Health knowledge has a significant effect on the adoption of health behaviors and attitudes in pandemic contexts.<br>Conclusions<br>The consolidation of these measures favors the consolidation of public rapid responses to infection outbreaks. The findings of this rapid review indicate a positive association between health knowledge and the adoption of health behaviors and attitudes. Therefore, a solid knowledge about categories of health would help people understand and adopt rapid responses to face a pandemic.
**Order of Authors:**	Fabio Alexis Rincon Uribe<br>Rejane Célia de Souza Godinho<br>Marcos Antonio Santos Machado<br>Kleber Roberto da Silva Gonçalves Oliveira<br>Cristian Ariel Neira Espejo<br>Natalia Carvalho Vianna de Sousa<br>Leonardo Lucas de Sousa<br>Marcos Vinicius Monteiro Barbalho<br>Pedro Paulo Freire Piani<br>Janari da Silva Pedroso
**Additional Information:**	

Powered by Editorial Manager® and ProduXion Manager® from Aries Systems Corporation

Question	Response
**Financial Disclosure**<br><br>Enter a financial disclosure statement that describes the sources of funding for the work included in this submission. Review the submission guidelines for detailed requirements. View published research articles from *PLOS ONE* for specific examples.<br><br>This statement is required for submission and **will appear in the published article** if the submission is accepted. Please make sure it is accurate.<br><br>**Unfunded studies**<br>Enter: *The author(s) received no specific funding for this work.*<br><br>**Funded studies**<br>Enter a statement with the following details:<br>• Initials of the authors who received each award<br>• Grant numbers awarded to each author<br>• The full name of each funder<br>• URL of each funder website<br>• Did the sponsors or funders play any role in the study design, data collection and analysis, decision to publish, or preparation of the manuscript?<br>• **NO** - Include this sentence at the end of your statement: *The funders had no role in study design, data collection and analysis, decision to publish, or preparation of the manuscript.*<br>• **YES** - Specify the role(s) played.<br><br>* typeset	The funders had no role in study design, data collection and analysis, decision to publish, or preparation of the manuscript.
**Competing Interests**<br><br>Use the instructions below to enter a competing interest statement for this submission. On behalf of all authors, disclose any competing interests that could be perceived to bias this work—acknowledging all financial support and any other relevant financial or non-financial competing interests.<br><br>This statement **will appear in the published article** if the submission is	The authors have declared that no competing interests exist.

accepted. Please make sure it is accurate. View published research articles from *PLOS ONE* for specific examples.<br><br>**NO authors have competing interests**<br><br>Enter: *The authors have declared that no competing interests exist.*<br><br>**Authors with competing interests**<br><br>Enter competing interest details beginning with this statement:<br><br>*I have read the journal's policy and the authors of this manuscript have the following competing interests: [insert competing interests here]*<br><br>* typeset	
**Ethics Statement**<br><br>Enter an ethics statement for this submission. This statement is required if the study involved:<br><br>• Human participants<br>• Human specimens or tissue<br>• Vertebrate animals or cephalopods<br>• Vertebrate embryos or tissues<br>• Field research<br><br>Write "N/A" if the submission does not require an ethics statement.<br><br>General guidance is provided below. Consult the submission guidelines for detailed instructions. **Make sure that all information entered here is included in the Methods section of the manuscript.**	N/A

**Format for specific study types**<br><br>**Human Subject Research (involving human participants and/or tissue)**<br>• Give the name of the institutional review board or ethics committee that approved the study<br>• Include the approval number and/or a statement indicating approval of this research<br>• Indicate the form of consent obtained (written/oral) or the reason that consent was not obtained (e.g. the data were analyzed anonymously)<br><br>**Animal Research (involving vertebrate animals, embryos or tissues)**<br>• Provide the name of the Institutional Animal Care and Use Committee (IACUC) or other relevant ethics board that reviewed the study protocol, and indicate whether they approved this research or granted a formal waiver of ethical approval<br>• Include an approval number if one was obtained<br>• If the study involved *non-human primates*, add *additional details* about animal welfare and steps taken to ameliorate suffering<br>• If anesthesia, euthanasia, or any kind of animal sacrifice is part of the study, include briefly which substances and/or methods were applied<br><br>**Field Research**<br><br>Include the following details if this study involves the collection of plant, animal, or other materials from a natural setting:<br>• Field permit number<br>• Name of the institution or relevant body that granted permission	
**Data Availability**<br><br>Authors are required to make all data underlying the findings described fully available, without restriction, and from the time of publication. PLOS allows rare exceptions to address legal and ethical concerns. See the PLOS Data Policy and FAQ for detailed information.	Yes - all data are fully available without restriction

A Data Availability Statement describing where the data can be found is required at submission. Your answers to this question constitute the Data Availability Statement and **will be published in the article**, if accepted.<br><br>**Important:** Stating 'data available on request from the author' is not sufficient. If your data are only available upon request, select 'No' for the first question and explain your exceptional situation in the text box.<br><br>Do the authors confirm that all data underlying the findings described in their manuscript are fully available without restriction?	
**Describe where the data may be found in full sentences. If you are copying our sample text, replace any instances of XXX with the appropriate details.**<br><br>• If the data are **held or will be held in a public repository**, include URLs, accession numbers or DOIs. If this information will only be available after acceptance, indicate this by ticking the box below. For example: *All XXX files are available from the XXX database (accession number(s) XXX, XXX.*).<br>• If the data are all contained **within the manuscript and/or Supporting Information files**, enter the following: *All relevant data are within the manuscript and its Supporting Information files.*<br>• If neither of these applies but you are able to provide **details of access elsewhere**, with or without limitations, please do so. For example:<br><br>*Data cannot be shared publicly because of [XXX]. Data are available from the XXX Institutional Data Access / Ethics Committee (contact via XXX) for researchers who meet the criteria for access to confidential data.*<br><br>*The data underlying the results presented in the study are available from (include the name of the third party*	All relevant data are within the manuscript and its Supporting Information files.

and contact information or URL).<br>• This text is appropriate if the data are owned by a third party and authors do not have permission to share the data.<br><br>* typeset	
Additional data availability information:	

Click here to access/download;Manuscript;Manuscript.docx

Effects of health knowledge in the health behaviors and attitudes adoption during pandemic emergencies: a rapid review

Health knowledge, health behaviors and attitudes during pandemics

Fabio Uribe [1*¶], Rejane Godinho[2¶], Marcos Machado[2¶], Kleber Oliveira[2], Cristian Espejo[2], Natalia Sousa[2&], Leonardo Sousa[2&], Marcos Barbalho[3&], Pedro Piani[4], Janari Pedroso[1,2]

[1] Programa de Pós-graduação em Psicologia, Universidade Federal do Pará, Belém, Brasil

[2] Programa de Pós-graduação em Teoria e Pesquisa do Comportamento, Universidade Federal do Pará, Belém, Brasil.

[3] Programa de Graduação em Psicologia, Universidade Federal do Pará, Belém, Brasil.

[4] Instituto de Ciências da Saúde, Universidade Federal do Pará, Belém, Brasil.

*Corresponding author

E-mail: faru1095@gmail.com

¶ These authors contributed equally to this work.

& These authors also contributed equally to this work.

Abstract

Background

People with good health knowledge present a conceptual and objective appropriation on general and specific health topics, increasing their probability to express health protection and prevention measures. The main objective of this study was to carry out a literature quick review about the effects of health knowledge on the adoption of health behaviors and attitudes in populations under pandemic emergencies.

Methods

A systematic review was performed according to the parameters of the PRISMA-P Group and the Cochrane method for rapid systematic reviews. Studies searches were performed in APA PsycNet, Embase, Cochrane Library and PubMed Central. Studies published between January 2009 and June 2020 and whose primary results reported a measure of interaction between health knowledge, health attitudes and behaviors in population groups during pandemics were included. A review protocol was recorded in PROSPERO (CRD42020183347).

Results

Out of a total of 5791 studies identified in the databases, 13 met the inclusion criteria. The included studies contain a population of 26099 adults, grouped into cohorts of health workers, university students, clinical patients, and the general population. Health knowledge has a significant effect on the adoption of health behaviors and attitudes in pandemic contexts.

Conclusions

The consolidation of these measures favors the consolidation of public rapid responses to infection outbreaks. The findings of this rapid review indicate a positive association between health knowledge and the adoption of health behaviors and attitudes. Therefore, a solid knowledge about categories of health would help people understand and adopt rapid responses to face a pandemic.

Introduction

Health knowledge is an objective construct that includes detailed and specific information on the etiology, prevalence, risk factors, prevention, transmission, symptomatology and disease treatment, as well as on health services and patient rights [1]. Previous evidence has demonstrated the positive effects of appropriate levels' health knowledge by community in general in health promotion and disease prevention [2,3]. Likewise, in the midst of public health emergencies, health knowledge reported by the public plays an important role in reducing risky behaviors and adopting protective and preventive practices [4,5]. However, in the broad empirical research in public health, a consistent and clear definition of health knowledge has not been offered1 and its lack of conceptual clarity has not allowed solid evidence that show its effects on behavior, or people's attitudes, or their contribution to the development of public policies [6]. In the pandemics' context, the literature evidence suggests that people who report high levels of knowledge about pandemics (definition, development, severity) linked to some aspects of the disease (transmission, prevention, causes) present an increase in the preventive measures practice, such as hand washing, the use of biosafety elements (masks, gloves, disinfectants), avoiding crowds, covering the face when coughing or sneezing, and seeking medical help in case of symptoms [7,8]. Likewise, adequate knowledge about pandemics and general health

information helps to correct misconceptions about circumstances related to pandemics, increasing the perception of susceptibility against the infection risk and improving the self-efficacy of self-protection [9,10]. However, there are some inconsistencies in the broad field of public health research during pandemic periods. The knowledge evaluation seems to focus on explicit and general information people have about knowledge's definition, causes and prevention of a pandemic, without investigating if this knowledge is linked, at the same time, with individual and public appropriation on the general and specific issues in health, associated with a personal commitment to take care of themselves and contribute to collective care [11,12]. Thus, the investigations have focused on the exclusively cross-sectional analysis of these results and have not explored if knowing about general, specific and objective health information could influence in the adoption of behaviors, practices or attitudes prevention and protection during the infection period and after that [13,14].

Previous evidence synthesis has addressed the knowledge role in behavioral responses during pandemics. A systematic review of studies published up to 2010 examined the demographic and attitudinal determinants of protective behaviors during a pandemic [15]. In their results, an association was identified between knowledge about the transmission of SARS, and the pandemic's meaning, and a greater execution of prevention behaviors, and intentions to comply with quarantine. Another studies review published between March 2009 and August 2011 analyzed the community response to the H1N1 influenza pandemic, particularly determining if these behaviors were related to the level of pandemic knowledge [16]. In their results, it is indicated that pandemic knowledge (transmission and prevention) is a factor that contributes to the adoption of preventive behaviors (hygiene behaviors, quarantine compliance, avoiding crowds and wearing a mask). Given that the previous reviews report the relevant role of knowledge about pandemics, our objective was to offer a quick synthesis of the evidence on the

effect of knowledge on health as a broader measure, that includes detailed and objective information on health components, in the adoption of health behaviors and attitudes during pandemics.

Why is this quick review important?

The promotion of health knowledge is a fundamental strategy for diseases control and the health maintenance in all populations. In public health emergencies, adequate health knowledge will help the broader community understand risk factors and generate quick responses to contain infections outbreaks [17]. In this way, in the midst of the public health emergency generated by COVID-19, political agents and health entities require synthesis of scientific evidence for the development of plans and strategies that contribute to the consolidation of preventive measures for the mitigation and control of massive outbreaks in all populations, especially those with a more precarious health system. These findings may offer an evidence-based explanatory framework that facilitates the early configuration of rapid behavioral and cognitive responses that guarantee the personal and collective care of communities in the context of pandemic emergencies. In this way, in response to the strategic objectives of the emergency committee, which seek to promote preventive measures and raise awareness of health issues in all populations in the current public emergency of COVID-19 [18], the World Health Organization recommends the execution of rapid reviews which results can strengthen health policies and systems [19].

Methods

This systematic review was carried out under the standards established in “Preferred Reporting Items for Systematic Reviews and Meta-Analyzes” (PRISMA-P) [20]. The Interim Guidance

from the Cochrane Rapid Reviews Methods Group was used as a methodological guide [21]. A review protocol was recorded in the International Prospective Registry of Systematic Reviews - PROSPERO (Record number: CRD42020183347). The PRISMA reporting guideline for systematic reviews was used to inform this work (S1 Appendix).

Eligibility criteria

The studies were included if their primary results reported a significant effect measure indicating the association degree between health knowledge and health behaviors or attitudes in pandemic situations (H1N1, Mers-Cov, COVID-19. In addition, articles published between January 2009 and June 2020 were included and limited to the English language only. The details of the inclusion and exclusion criteria are available in S1 Table.

Search Strategy

Systematic literature searches were performed on APA PsycNet (American Psychological Association), Embase, Cochrane Library, and PubMed Central. The search criteria included controlled vocabulary MeSH (Medical Subject Headings) on *Health Awareness*, *Health Knowledge*, *Pandemic*, *Health Behavior* and *Health Attitudes*. The search strategy was developed and adapted for all data-bases. The complete search strategy is available in S2 Appendix.

Study selection and data extraction

All references were imported into Mendeley software to manage data and eliminate duplicates. Three authors independently screened abstracts and full-texts of potential studies using the inclusion and exclusion criteria and any discrepancies were discussed in group. Data were extracted using a standardized extraction form, and the compilers indicated information about

author(s), year, country, design, participant characteristics and findings (primary results and measure of association). For the measures of association, linear dependence indicators (correlation coefficients), odds ratio (Odds Ratio), standardized coefficients and confidence intervals were considered. Likewise, statistical significance tests were included as solid evidence of the reported findings. The data was extracted by one author with complete verification of two authors.

Quality assessment

The quality assessment was conducting by one author (FR), with complete verification of JP and KS, using a modified version of the Ottawa – Newcastle scale (NOS) for cross sectional studies [22]. The NOS contains a checklist consisting of three criteria: selection (representativeness of individuals); comparability (determination of confusion) and result (evaluation and analysis of results). According to the scoring system, studies are rated in a range of 0 to 10 points and are classified as low (10 and 9 points), medium (7 and 8 points) high (<7 points). In general, the NOS scale has presented good reliability between evaluators and test-retest [23].

Synthesis and analysis

In this rapid review, findings were presented and synthesized using a narrative and thematic synthesis approach. The synthesis presents comments and interpretations of the extracted data, including the interaction effects between the variables described in the section on the information collection and extraction process. In this way, the categories of analysis were structured considering the effect of health knowledge on the adoption of health behaviors and attitudes during pandemic.

Results

Searching the online databases identified 5791 publications. Also, 5 additional studies were found using manual searches that included results of interest. After articles duplicates' exclusion and the selection by abstracts and titles, 92 articles were chosen for full-text selection. Of this total, 79 did not meet the proposed inclusion criteria, leaving 13 eligible articles. The complete study selection process is presented in Fig 1.

Fig 1. PRISMA flow diagram of the study selection process.

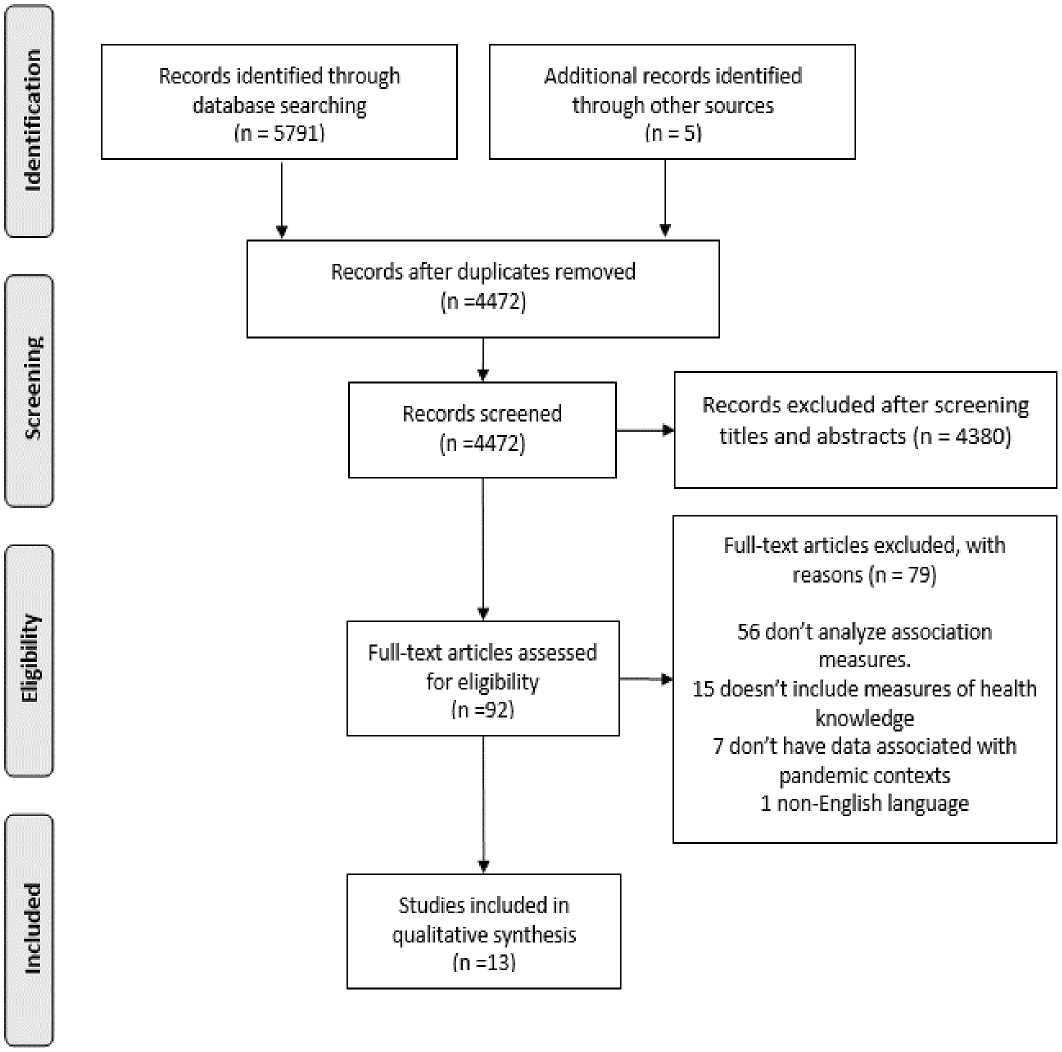

Studies' Characteristics

The 13 eligible studies examined the relationship between health knowledge and health behaviors or attitudes during pandemics, with a publication date that varies between 2009 and 2020. 13 different population cohorts were analyzed in countries such as Bangladesh, China, Egypt, Hong Kong, India, Iran, Malaysia, United States, Saudi Arabia and Singapore. A total of 26099 peoples were interviewed, grouped into cohorts of health workers (n = 2683), university students (n = 4194), clinical patients (n = 3160), and the general population (n = 16062). All studies reported cross-sectional data analyzes in three pandemic settings: Mers-CoV, Influenza A H1N1, and COVID-19. The characteristics and primary results of the studies were extracted and summarized in Table 2.

Methodological quality assessment

The quality evaluation description for each criterion, as well as the general score is available in S2 Table. Overall, 8 studies had a moderate risk of bias [24-31], while 5 studies showed a high risk of bias [32-36]. The sample was representative in 4 of the thirteen studies [24-26,30] but only in 3 studies was the random process selection of the sample described [25,26,30]. All studies used self-report measures, however, only the authors of 7 studies presented the instruments validation [25,27,29-31,33,35].

Table 2. Characteristics and primary studies result.

Author(s), year and country	*Design*	*Sample*	*Findings*		*Bias Risk*
			Primary Outcome	*Measure of Association*	
Almutairi et al., 2015, Saudi Arabia	Cross-sectional	1147 adults aged >18 years. Male 62%, female 38%.	Health knowledge was a significant predictor for precautionary practices and attitudes in the coronavirus pandemic (MERS-CoV).	Behaviors: $\beta = 0.28$; *SE = 0.02*; $p < 0.001$.<br>Attitudes: $\beta = 0.35$; *SE = 0.08*; $p < 0.001$.	High
Askarian et al., 2013, Iran	Cross-sectional	125 resident doctors. The mean age was 30,62 (SD= 5.17) years. Male 59,2%, female 40,8%.	Health knowledge was positively and significantly correlated with health protection practices for pandemic H1N1 influenza.	*r = 0,45; p value <0.001*	Average
Etingen et al., 2013, United State	Cross-sectional	3113 veterans. The mean age was 61,82 (SD=11.70). Male 96,97%, female 3,03%.	An adequate reception of health information during H1N1 influenza increased the probability of presenting self-protective behaviors in a cohort of older adults	Wear a facemask OR = 1.39, 95 % CI 0.99 1.95, $p = 0.053$.<br><br>Stay home to avoid illness' OR = 0.69, 95 % CI 0.53–0.90, $p = 0.006$.	High
Ho et al., 2013, Singapore	Cross-sectional	1055 adults aged > 18 years. Male 45,3% and female 54,7%.	Public health knowledge was positively associated with precautionary behavior intentions in the H1N1 pandemic.	$\beta = 0.11$, $p < 0.001$	Average
Keller et al., 2014, China.	Cross-sectional	2882 university students. Age not reported. Male 70.4%, female 66.6%.	Health knowledge about H1N1 moderately predicted preventive health behaviors.	Wear a facemask: OR = 0.99, 95% IC 0.81 – 1,20<br>Increased Hand Washing: OR = 1,10, 95% IC 0.94 – 1.30<br>Reporting Symptoms OR = 1.10, 95% IC 0.92 – 1.33.	High

Krishnappa et al., 2020, India	Cross-sectional	860 dentists. Continent: Asia 30.7%, Americas 25%, Europe 16.3%, Africa 22.6% and other (Australia and Antarctica 22.6%).	Health knowledge was significantly associated with protective practices in the COVID-19 pandemic.	$r = 0.669$; 95% CI 0,77-26,64 $p < 0.05$	Average
Liao et al., 2010, Hong Kong	Cross-sectional	1016 adults aged >18 years. Male 46%, female 54%.	Health knowledge is a partial mediator between confidence in formal information and personal hygiene practices towards the Influenza A (H1N1) pandemic.	$\beta = 0,35$; ~17% mediation.	Average
Lin et al., 2011, China	Cross-sectional	10669 adults. Aged between 18 and 90 years (M = 41.47 years). Male 45.6%, female 54.4%.	Health knowledge was significantly associated with self-protection practices during the H1N1 pandemic.	OR =1.57; 95% CI, 1.42 to 1.73; $p<0.0001$	Average
Nabil et al., 2011, Egypt	Cross-sectional	1312 university students. Male 50.3%, female 49.7%.	Health knowledge in university students was associated with more availability to comply with home quarantine during the H1N1 pandemic.	OR = 0.27, 95% CI 0.2 - 0.34, $p < 0.001$.	Average
Ping et al., 2011, Malaysia	Cross-sectional	1049 adults aged between 18 and 19 years. Male 37%, female 63%. Malay 41%, Indian 25% and Chinese 34%.	Health knowledge was a significant predictor for the health protective behaviors practice in the three ethnic groups for the pandemic H1N1 outbreak.	Malay: $\beta = 0.08$; $SE = 0.03$; $p < 0.05$; Chinese: $\beta = 0.10$; $SE = 0.03$; $p < 0.001$; Indian: $\beta = 0.08$; $SE = 0.03$; $p < 0.01$.	Average

Rahman et al., 2020, Bangladesh	Cross-sectional	441 adults. 85,7% aged between 18 and 29 years, and 14.3% aged in 30 years or more. Male 68.7% and female 31.3%.	Health knowledge about COVID-19 increases the likelihood of executing preventive practices such as wearing masks and staying home.	Wear a facemask: AOR =1.54; 95% CI, 1.25 to 1.77; p<0.01.<br>Stay home: AOR=1.73; 95% CI, 1,43 to 2,09; p<0.01.	High
Yap et al., 2010, Singapore	Cross-sectional	1063 adults aged between 17 and 61 years (M=21.4; SE=0.2). Male 95.8%, female 4.2%. Chinese 75.6%, Malay 13.5%, Indian 5.8% and others 2.9%.	High health knowledge was a significant predictor for high levels of protection practices and attitudes towards the Influenza A (H1N1) pandemic.	Practices: β =0.30; 95% CI 0.22 – 0.37; p < 0.001.<br>Attitudes β =0.21; 95% CI 0.14 – 0.28; p < 0.001.	Average
Zhang et al., 2020, China	Cross-sectional	1367 health workers. Male 53.4%, female 46.7%. Doctors 36.5%, Nurses 46.5% and Paramedics 17%	Health knowledge significantly influenced the protective attitudes of health workers.	Attitudes: “Confidence in defeating the virus”, OR = 1.41, 95% CI 1.12 - 1.77, p < 0.01.). “Patients must disclose their exposure” OR = 1.217, 95% CI 1.04 – 1.42, p < 0.001.	High

195 β = beta coefficient, SE = Standard Error, OR: Odds Ratio, AOR = Adjusted Odds Ratio, CI = Confidence Interval

Measure of health knowledge

In all the studies, the measure of health knowledge was quantified through self-report instruments (surveys and questionnaires), and detailed and objective information on the health measures applicable in the pandemics' context was included. Health knowledge was evaluated as a continuous measure and operationalized through categories focused on the disease or the virus spread during pandemics, such as: transmission [24-36], prevention [24,25,28-36], symptomatology [24,26,27,29-31,33-35], definition of the pandemic [24,31,34,36], vaccine availability [28-30,33], infection severity [26,27,29,30], etiology [25,29,34], incubation period [29,34] and communicability period [34]. The frequencies of each category are available in S3 Table.

Health knowledge and health behaviors

A total of 13 studies examined the effect of health knowledge on the adoption of health behavior during pandemics. The authors of 4 studies found that health knowledge was positively associated [28-31] with health behaviors linked with hygiene practices, use of biosecurity elements, self-protection behaviors and social distancing. Likewise, two studies found that health knowledge behaved as a significant predictor for the execution of protective behaviors [24] and precautionary behavior [34], such as: hand washing, wearing a mask, covering up when sneezing or coughing, monitoring the temperature, avoiding public places and seeking medical help. In one study [27] it was found that health knowledge predicted protective behaviors in three different ethnic groups: Malay, Chinese, and Indian. However, one study found that health knowledge presents a moderate prediction towards preventive behaviors such as the use of masks, hand washing and symptom report [33].

Furthermore, two studies found that health knowledge increases the probability of executing preventive practices (wear a facemask and stay home to avoid illness') both in clinical contexts [32] as in non-clinical settings [35]. Also, in one study it was reported that health knowledge favored compliance with home quarantine [26]. In relation to the role that health knowledge played in the awareness processes exercised by the government through the pandemic H1N1 emergency, one study reported that health knowledge functioned as a significant mediator between reliance on formal health information and the adoption of hygiene practices such as: hand washing, covering face when sneezing or coughing, and disposing items used to sanitize objects [25].

Health knowledge and health attitudes

Three studies explored the influence of health knowledge on the acquisition of health attitudes. The authors found that health knowledge acted as a predictor of attitudes associated with health issues during pandemics [24,34,36]. Yap et al. (2010) [24] associated the levels of knowledge (definition, transmission, prevention, and symptoms) and attitudes related to preventive measures in a cohort of adults during the H1N1 pandemic. It was observed that high levels of health knowledge in the participants allowed them to have better attitudes, reflecting adequate perceptions about the implementation of hygiene and self-protection practices, the importance of influenza vaccination, the role of medical services and the risk of infection.

For their part, Almutairi et al., (2015) [34] examined the influence of knowledge on health and attitudes related to the coronavirus pandemic (MERS-CoV). The authors found significant predictive values that linked the health knowledge of a group of Saudi adults with more adequate attitudes towards the pandemic. These were related to correct perceptions about the severity of

the disease, the health measures proposed by the government and the availability to comply with social restrictions. Finally, Zhang et al., (2020) [36] explored the link between health knowledge and health attitudes in a group of health workers facing the COVID-19 pandemic. The findings allowed observing that high levels of health knowledge in health workers favored their attitudes towards the COVID-19 pandemic emergency.

Discussion

This systematic review identified 13 eligible studies that evaluated measures of knowledge, behavior, and health attitudes in adult cohorts during pandemic outbreaks of MERS-CoV, H1N1, and COVID-19. The synthesis result of the evidence suggests that health knowledge has a significant effect on the acquisition of health behaviors and attitudes, reflecting the efforts of the health system, the government and the community to face the negative impact of pandemic emergencies. Thus, these results show the predictive and mediating role of health knowledge in adopting preventive and self-protective health behaviors and attitudes, which help to consolidate rapid responses, more effective, by the public, against the risk of pandemic infection.

In all the studies, health knowledge was approached as a continuous measure, where the information that the participants had regarding health issues in pandemic situations was evaluated. The wide conceptual heterogeneity of the health knowledge variable was evident in the synthesis of the results, however, most of the studies included central categories: transmission, symptoms, etiology, infection severity and prevention [29,30,34]. Although in public health research, the application of undifferentiated and arbitrary meanings to the term health knowledge has generated conceptual confusions that represent a significant source of bias [1], from the findings of this review it is evident that health knowledge has been approached from

categories that report the characteristics of the disease (definition, etiology, transmission, prevention, symptoms), excluding positive health factors, such as: lifestyles, healthy habits, protective factors, rights and provision of health services. A less pathological approach and with a greater emphasis on positive health aspects could broaden the notions of the health of the public and its effect of interaction with changes in people's behavior could bring beneficial effects for the public health of society.

The effect of health knowledge on the adoption of health behavior was explored in most of the studies. The results indicate that health knowledge is a predictor and mediator of hygiene practices, self-protection behaviors, restriction and social distancing or seeking medical help in all cohorts in the midst of pandemic contexts. Four were the health behaviors with the highest prevalence in the study’s general synthesis: hand washing, mask’s use, covering up the face when sneezing or coughing, and social isolation, finding an increase in the execution of these practices when the participants reported good knowledge in health [24,28]. This interaction effect between health knowledge and changes in people's behavior can be explained by the practical nature, easy access and simplicity in the execution of these practices, which could facilitate their adherence to lifestyles and participants' individual hygiene habits. During the early stages of pandemic outbreaks (H1N1 and Covid-19), people who reported high levels of health knowledge on aspects such as transmission, symptomatology, and prevention adopted these measures as rapid responses for control and surveillance of outbreaks massive infection [25,27,35]. Previous evidence has shown that the timely implementation of these practices significantly reduces the spread of highly pathogenic viruses [37,38].

Only three studies found a significant effect of health knowledge on participants' health attitudes. All three studies provide some evidence that health knowledge may be a predictor of health

attitudes during pandemic outbreaks [24,34,36]. Furthermore, the evidence suggests that adequate health knowledge helps correct misconceptions and myths about the disease, as well as increasing the perception of susceptibility against the infection risk [24,34]. These attitudes were characterized as being intentional, evaluative, subjective, and cognitive in nature, reflecting perceptions and thoughts about government measures to contain outbreaks, the risk of infection, and evaluative judgments about exposure to the disease. These findings may explain the effect of acquiring correct health information and the selection of legitimate information sources for an adequate understanding of the circumstances of a pandemic emergency, especially those associated with health care and prevention [39].

Limitations

To consider the evidence provided in this rapid review, is important to take account of its limitations. Initially, the cross-sectional nature of all the studies may limit the validity and generalization of the findings. Second, a significant heterogeneity of the data was found, due to the methodological and conceptual variation used in the studies. The reasons that could explain these limitations is the challenge of obtaining reliable estimates, with a high degree of evidence, in the context of a pandemic. In this way, the non-random sampling of the sample, the use of self-report scales and questionnaires, and the use of virtual means for the measurement of the variables can represent an important source of bias. However, this rapid review of the evidence found a substantial number of studies that empirically substantiate the effects of health knowledge on health behaviors and attitudes during public health emergencies caused by pandemics.

Conclusions

The findings of this rapid review indicate an association between health knowledge and the adoption of health behaviors and attitudes. However, the data nature to examine the effects of their interaction remains challenging in pandemic settings. The concept of health knowledge needs to be extrapolated from mere pandemic knowledge and must incorporate clear and detailed information on general and specific aspects of health, linked to adequate values, intentions and perceptions about personal and collective care in the midst of pandemics. Therefore, a clear, consistent and reliable knowledge on health issues would help people to understand and adopt rapid behavioral and cognitive responses to face a pandemic. Also, would allow the development of healthy public policies, based on communities' experience that highlight the capacity for people commitment and the actions' efficiency of health promoters, researchers and government entities to mitigate risks and harmful factors for health. On the other hand, an adequate conceptual construction could be of utmost importance to address the contribution of health knowledge in emergency situations. Thus, for future research, one could try to adopt general and specific categories in health, including aspects associated with health care and disease prevention, in order to guarantee the homogeneity and generalization of empirical results. Likewise, could favor the adequate transmission of health information, also representing an effective strategy to control the massive spread of highly pathogenic viruses.

References

1. Trevethan R. Deconstructing and Assessing Knowledge and Awareness in Public Health Research. *Front Public Health* 2017; 5: 1-6. doi: 10.3389/fpubh.2017.00194

2. Harrell T, Davy B, Stewart J, King D. Effectiveness of a school-based intervention to increase health knowledge of cardiovascular disease risk factors among rural Mississippi

middle school children. *South Med J* 2005; 98: 1173 - 80. doi: 10.1097/01.smj.0000182499.59715.07

3. Khaled G, Badar S, Ahmed A, Al-Shaikh R, Al-Mussaed E, Habib F, et al. Effectiveness of health education programme: Level of knowledge about prevention of cervical cancer among Saudi female healthcare students. *J Pak Med Assoc* 2017; 67: 513 – 21. PMID: 28420907

4. McCallum J, Arekere D, Lee B, Katz R, Rivers B. Awareness and knowledge of the U.S. Public Health service syphilis study at Tuskegee: implications for biomedical research. *J Health Care PooR Underserved* 2016; 17: 716 - 33. Doi: 10.1353/hpu.2006.0130

5. Manabe T, Thuy T, Manh D, Hong D, Phuong P, Thanh D, et al. Knowledge, attitudes, practices and emotional reactions among residents of avian influenza (H5N1) hit communities in Vietnam. *PLoS One* 2012; 7: e47560. Doi: 10.1371/journal.pone.0047560

6. Bryant T. Role of knowledge in public health and health promotion policy change. *Health Promot Int* 2002; 17: 89 – 98. Doi: 10.1093/heapro/17.1.89

7. Agüero F, Nebot M, Pérez A, López M, Garcia X. Adoption of preventive measures during and after the 2009 influenza A (H1N1) virus pandemic peak in Spain. *Prev Med* 2011; 53: 203 – 06. Doi: 10.1016/j.ypmed.2011.06.01

8. Janagan E, Hariharan S. Public health awareness: knowledge, attitude and behaviour of the general public on health risks during the H1N1 influenza pandemic. *J Public Health* 2017; 25: 333-37. Doi: 10.1007/s10389-017-0790-7

9. Fai J, Griffiths S, Chow K, Yi H. Widespread public misconception in the early phase of the H1N1 influenza epidemic. *J Infec* 2009; 59: 122 - 27. Doi: 10.1016/j.jinf.2009.06.004

10. Ping L, Ching I. Knowledge and Attitudes in Regard to Pandemic Influenza A (H1N1) in a Multiethnic Community of Malaysia. *Int J Behav Med* 2011*; 18: 112 - 21.* Doi: 10.1007/s12529-010-9114-9

11. Naing C, Yi R. Knowledge about the pandemic influenza A (H1N1) and willingness to accept vaccination: a cross-sectional survey. *J Public Health* 2011; 19: 511 – 16. Doi: 10.1007/s10389-011-0434-2

12. Srikanth A, Ali W, Rahmani J, Ashrafi M, Kumar D. Knowledge and perceptions of COVID-19 among health care workers: cross-sectional study. *JMIR Public Health Surveill* 2020; 6: e19160. Doi: 10.2196/19160

13. Akan H, Gurol Y, Izbirak G, Ozdatli S, Yilmaz G, Vitrinel A, et al. Knowledge and attitudes of university students toward pandemic influenza: a cross-sectional study from turkey. *BMC Public Health* 2010; 10: 1 – 8. Doi: 10.1186/1471-2458-10-413

14. Puri S, Singh A, Koushal V, Thakare M, Singhal A. Knowledge, attitude and practice regarding the H1N1 pandemic amongst healthcare providers and preparedness in a multispecialty teaching hospital in North India. *Public Health* 2011, 125: 795 – 98. Doi: 10.1016/j.puhe.2011.09.004

15. Bish A, Michie S. Demographic and attitudinal determinants of protective behaviours during a pandemic: A review. *Br J Health Psychol* 2010; 15: 797-824. Doi: 10.1348/135910710X485826

16. Tooher R, Collins J, Street J, Braunack A, Marshall H. Community knowledge, behaviours and attitudes about the 2009 H1N1 Influenza pandemic: a systematic review. *Influenza and Other Respiratory Viruses* 2013; 7: 1316 - 27. Doi: 10.1111/irv.12103

17. Balkhy H, Abolfotouh M, Al-Hathlool R, Al-Jumah M. Awareness, attitudes, and practices related to the swine influenza pandemic among the Saudi public. *BMC Infec Dis* 2010; 10: 1- 7. Doi: 10.1186/1471-2334-10-42

18. World Health Organization. Coronavirus disease 2019 (COVID-19) – Situation report 5. 2020. https://apps.who.int/iris/handle/10665/331475

19. World Health Organization. Rapid reviews to strengthen health policy and systems: a practical guide. 2017. https://www.who.int/alliance-hpsr/resources/publications/rapid-review-guide/en/

20. Moher D, Shamseer L, Clarke M, Ghersi D, Liberati A, Petticrew M, *et al*. Preferred reporting items for systematic review and meta-analysis protocols (PRISMA-P) 2015 statement. *Syst Rev* 2015; 4: 1-9 . Doi: 10.1186/2046-4053-4-1

21. Garritty C, Gartlehner G, Kamel C, King V, Nussbaumer-Streit B, Stevens A, et al. Cochrane Rapid Reviews. Interim Guidance from the Cochrane Rapid Reviews Methods Group. March 2020.

22. Herzog R, Álvarez J, Díaz C, Del Barrio J, Estrada J, Gil A. Are healthcare workers´ intentions to vaccinate related to their knowledge, beliefs, and attitudes? A systematic review. *BMC Public Health* 2013; 13: 1 - 17. Doi: 10.1186/1471-2458-13-154.

23. Oremus M, Oremus C, Hall G, McKinnon M. Inter-rater and test-retest reliability of quality assessments by novice student raters using the Jadad and Newcastle-Ottawa Scales. *BMJ Open* 2012; 2: e001368. Doi: 10.1136/bmjopen-2012-001368

24. Yap J, Vernon J, Yan T, Pin T, Chem P. Knowledge, attitudes and practices towards pandemic influenza among cases, close contacts, and healthcare workers in tropical Singapore: a cross-sectional survey. *BMC Public Health* 2010; 10: 1-10. Doi: 10.1186/1471-2458-10-442

25. Liao Q, Cowling B, Wing W, Fielding R. The influence of social-cognitive factors on personal hygiene practices to protect against influenzas: using modelling to compare avian a/h5n1 and 2009 pandemic A/H1N1 Influenzas in Hong Kong. *Int J Behav Med* 2011; 18: 93 – 104. Doi: 10.1007/s12529-010-9123-8

26. Nabil N, Edward A. Knowledge, attitude and practice of El-Minia university students towards pandemic H1N1, Egypt, 2009. *J Public Health* 2011; 19: 505 – 10. Doi: 10.1007/s10389-011-0427-1

27. Ping L, Ching I. Behavioral responses to the influenza A(H1N1) outbreak in Malaysia. *J Behav Med* 2011; 34: 23 – 31. Doi: 10.1007/s10865-010-9283-7

28. Lin Y, Huang L, Nie S, et al. Knowledge, Attitudes and Practices (KAP) related to the Pandemic (H1N1) 2009 among Chinese General Population: a Telephone Survey. *BMC Infect Dis* 2011; 11: 1 – 9. Doi: 10.1186/1471-2334-11-128

29. Askarian M, Danaei M, Vakili V. Knowledge, attitudes, and practices regarding pandemic H1N1 influenza among medical and dental residents and fellowships in Shiraz, Iran. *Int j prev Med* 2013; 4: 396 – 403.

30. Ho S, Peh X, Soh V. The Cognitive Mediation Model: Factors Influencing Public Knowledge of the H1N1 Pandemic and Intention to Take Precautionary Behaviors. *J Health Commun* 2013; 18: 773 – 94. Doi: 10.1080/10810730.2012.743624

31. Krishnappa S, Sharma S, Thakar S, et al. Assessing Knowledge, Attitudes and Practices of dental practitioners regarding the COVID-19 pandemic: A multinational study. *Dent Med Probl* 2020; 57: 11 – 7. Doi: 10.17219/dmp/119743

32. Etingen B, La Vela S, Miskevics S, Goldstein B. Health Information During the H1N1 Influenza Pandemic: Did the Amount Received Influence Infection Prevention Behaviors? *J Community Health* 2013; 38: 443 – 50. Doi: 10.1007/s10900-012-9647-8

33. Keller J, Kim J, Lau J, Wong A, Griffiths S. Intention to engage in preventive behaviors in response to the a/h1n1 pandemic among university entrants in four Chinese cities. *Asia Pac J Public Health* 2014; 26: 42 – 7. Doi: 10.1177/1010539513496842

34. Almutairi K, Helih E, Moussa M, Boshaiqah A, Saleh A, Vinluan J, et al. Awareness, attitudes and practices related to coronavirus pandemic among public in Saudi Arabia. *Fam Community Health* 2015, 38: 332 – 40. Doi: 10.1097/FCH.0000000000000082

35. Rahman A, Sathi J. Knowledge, Attitude, and Preventive Practices toward COVID-19 among Bangladeshi Internet Users. *Electron J Gen Med* 2020; 17: em245. Doi: 10.29333/ejgm/8223

36. Zhang M, Zhou M, Tang F. Knowledge, attitude, and practice regarding COVID-19 among healthcare workers in Henan, China. *J Hosp Infect* 2020; 105: 183 – 87. Doi: 10.1016/j.jhin.2020.04.012

37. Aiello A, Perez V, Coulborn R, Davis B, Uddin M, Monto A. Facemasks, Hand Hygiene, and Influenza among Young Adults: A Randomized Intervention Trial. *PLoS One* 2012, 7: e29744. Doi: 10.1371/journal.pone.0029744

38. Suess T, Remschmidt C, Schink S, et al. The role of facemask and hand higiene in the prevention of influenza transmisión in households: results from a cluster randomised trial; Berlin, Germany, 2009 – 2011. *BMC Infections Diseases* 2012, 12: 2 -16. Doi: 10.1186/1471-2334-12-26

39. Weintraud A, Pinkleton B, Austin B, Van de Vord R. The relationships of information efficacy and media literacy skills to knowledge and Self-efficacy for health-related decisión making. *J Am Coll Health* 2012; 60: 548 – 54. Doi: 10.1080/07448481.2012.726302

Supporting information

S1 Appendix. PRISMA checklist

S2 Appendix. Search Criteria

S1 Table. Inclusion and exclusion criteria for article selection

S2 Table. Quality assessment of included studies

S3 Table. Operationalization of the health knowledge measurement

Click here to access/download
Supporting Information
S1 Appendix. PRISMA checklist.doc

Click here to access/download
Supporting Information
S2 Appendix. Search criteria.docx

Click here to access/download
Supporting Information
S1 Table. Eligibility criteria.docx

Click here to access/download
Supporting Information
S2 Table. Quality assessment of included studies.docx

Click here to access/download
Supporting Information
S3 Table. Health knowledge measurement.docx
